# Proteomic dataset for decellularization of porcine auricular cartilage

**DOI:** 10.1186/s13104-024-06716-9

**Published:** 2024-02-27

**Authors:** Roxanne N. Stone, Xinzhu Pu, Julia Thom Oxford

**Affiliations:** 1 Biomedical Engineering Doctoral Program, Boise State University, 83725 Boise, ID USA; 2 Biomolecular Research Center, Boise State University, 83725 Boise, ID USA; 3 Center of Biomedical Research Excellence in Matrix Biology, Boise State University, 83725 Boise, ID USA; 4 Biomolecular Sciences Graduate Program, Boise State University, 83725 Boise, ID USA; 5 Department of Biological Sciences, Boise State University, 83725 Boise, ID USA

**Keywords:** Osteoarthritis, Porcine, Cartilage, Decellularization, Proteomics, LC-MS/MS

## Abstract

**Objectives:**

Osteoarthritis (OA) is a major concern in the United States and worldwide. Development and validation of robust decellularization techniques is critical in generating suitable bioscaffolds for future OA treatment options.

**Data descriptions:**

In the present study, proteins from porcine auricular cartilage before and after decellularization were extracted, digested, and identified using liquid chromatography-tandem mass spectrometry (LC-MS/MS). The data represents protein profiles of both non-decellularized and decellularized porcine auricular cartilage. This data is intended to be useful to scientists who are interesting in generating biomaterials for potential relevant clinical applications using decellularized cartilage tissue.

## Objective

Osteoarthritis (OA) is one of the leading causes of disability worldwide [1, 2]. Tissue engineering approaches using 3-dimensional scaffolds are promising for the early treatment of cartilage degeneration in OA joints [3]. Development and validation of robust decellularization techniques is critically important in generating suitable scaffolds to provide support for tissue growth. Our dataset comprises quantitative proteomic analysis of porcine auricular cartilage before and after decellularization. We believe that this data would be beneficial for researchers who are interested in generating biomaterials using decellularized cartilage as a future alternative treatment option for individuals suffering from OA.

## Data description

This is a raw data set of our research article presenting our findings on creating and validating biological scaffold from porcine auricular cartilage using a decellularization protocol developed in our lab [4]. We performed decellularization using a combination of chemical and physical methods. Surfactants, acid and bases, and enzymes were included in the chemical and enzymatic treatment to remove cells [5–7]. Proteins from nondecellularized and decellularized scaffolds were digested with trypsin and the resulting peptide were chromatographically separated on a reverse-phase C18 column analyzed on a Linear Ion Trap mass spectrometer using a Data Dependent Acquisition workflow [4]. Peptide spectral matching was performed by a database search using Sequest HT algorithms in a Proteome Discoverer 2.2 (Thermo Fisher Scientific). Raw spectrum data were searched against the UniProtKB/Swiss-Prot protein database for *Sus scrofa* (May 25, 2019). Dataset includes raw data files and peak list files **(**Table 1**)** [8].

**Table 1:** Overview of data files/data sets

Label	Name of data file/data set	File types (file extension)	Data repository and identifier (DOI or accession number)
Data file 1	DecellularizedRawData	Raw data (.raw)	MassIVE (10.25345/C5HQ3S890) [8]
Data file 2	NondecellularizedRawData	Raw data (.raw)	MassIVE (10.25345/C5HQ3S890) [8]
Data file 3	DecellularizedPeaklist	Peak list (.mzML)	MassIVE (10.25345/C5HQ3S890) [8]
Data file 4	NondecellularizedPeaklist	Peak list (.mzML)	MassIVE (10.25345/C5HQ3S890) [8]

## Limitations

- Current data is of scaffolds generated from porcine auricular cartilage and may differ from biomaterials generated from decellularization of other tissues.
- The data is generated using a linear ion trap mass spectrometer and thus the mass resolution is slightly less compared to other high-resolution platforms like Orbitrap data.

## Data Availability

Proteomic dataset has been deposited in MassIVE repository and is available at: https://doi.org/10.25345/C5HQ3S890.

## Abbreviations

OA: Osteoarthritis
LC-MS/MS: Liquid chromatography-tandem mass spectrometry
